# Control of Glucosylceramide Production and Morphogenesis by the Bar1 Ceramide Synthase in *Fusarium graminearum*

**DOI:** 10.1371/journal.pone.0019385

**Published:** 2011-04-29

**Authors:** William R. Rittenour, Ming Chen, Edgar B. Cahoon, Steven D. Harris

**Affiliations:** 1 Department of Plant Pathology, University of Nebraska-Lincoln, Lincoln, Nebraska, United States of America; 2 Department of Biochemistry, University of Nebraska-Lincoln, Lincoln, Nebraska, United States of America; 3 Center for Plant Science Innovation, University of Nebraska-Lincoln, Lincoln, Nebraska, United States of America; University of Wisconsin–Madison, United States of America

## Abstract

The contribution of plasma membrane proteins to the virulence of plant pathogenic fungi is poorly understood. Accordingly, the objective of this study was to characterize the acyl-CoA dependent ceramide synthase Bar1 (previously implicated in plasma membrane organization) in the wheat pathogen *Fusarium graminearum*. The role of Bar1 in mediating cell membrane organization was confirmed as Δ*BAR1* mutants failed to display a distinct sterol-rich domain at the hyphal tip. The Δ*BAR1* mutants were non-pathogenic when inoculated onto wheat heads, and their *in vitro* growth also was severely perturbed. Δ*BAR1* mutants were incapable of producing perithecia (sexual fruiting structures) and only produced macroconidia (asexual spores) in the presence of NaCl. Sphingolipid analyses indicated that Bar1 is specifically necessary for the production of glucosylceramides in both *F. graminearum* and *Aspergillus nidulans*. Interestingly, glucosylceramides appear to mediate sensitivity to heat stable antifungal factor (HSAF), as, in addition to Δ*BAR1* mutants, a glucosylceramide synthase deficient mutant of *Yarrowia lipolytica* is also resistant to HSAF.

## Introduction

*Fusarium graminearum* (teleomorph *Gibberella zeae*) is the causative agent of head blight (scab) on small grains such as wheat. The effects of the disease are two-fold; infected plants produce less grain, and the grain that is produced can contain various mycotoxins, particularly deoxynivalenol [1], [2]. The significant economic impact of head blight in wheat-growing areas of the United States and Europe has triggered interest in understanding the mechanisms that underlie interactions between *F*. *graminearum* and prospective host plants [3], [4]. Of the several features distinguish *F*. *graminearum* from the well-characterized *Magnaporthe oryzae*-rice pathosystem, the most notable is the absence of appressoria production by *F. graminearum* during host invasion [1]. Accordingly, the identification and characterization of functions required for the pathogenicity of *F*. *graminearum* should provide a broader perspective on virulence mechanisms deployed by fungi.

We are interested in exploring the idea that lipid microdomains located on the surface of fungal plant pathogens play an important role in host interactions. In animals and yeast, signaling complexes have been shown to aggregate into lipid microdomains termed “lipid rafts” [5], [6]. These rafts are regions of the plasma membrane rich in sterols and sphingolipids, which alter the biochemical properties of the domains and confer resistance to mild detergents [7], [8]. In fungi, several proteins have been isolated from detergent-resistant membrane (DRM) domains in *Saccharomyces cerevisiae, Candida albicans,* and *Cryptococcus neoformans.* A general trend for DRM proteins appears to be the presence of a glycosylphosphatidylinositol (GPI) anchor, the lipid tail of which is presumed to interact favorably with saturated sphingolipids [9], [10]. However, many transmembrane proteins are also found to be enriched in DRM fractions [11]. For example, the ATPase Pma1 has become a marker for DRM fractions in both *S. cerevisiae* and *C. albicans* [8], [9]. Given their involvement in clustering membrane proteins and signaling complexes on the cell surface, lipid rafts represent attractive targets for the discovery of novel virulence factors in plant pathogenic fungi (such as those that might regulate MAP kinase and other signaling pathways; [12]). Consistent with this notion, GPI-anchored virulence determinants Sod1 (Cu/Zn superoxide dismutase) and Plb1 (lysophospholipase) localize to DRM fractions of the human pathogen *C. neoformans* [13].

Sterol-rich domains are crucial for polarized hyphal growth in a number of fungi, as disruption of their organization typically results in failure to maintain a discrete polarity axis. The importance of these domains has been demonstrated by the genetic characterization of several *A. nidulans* mutants affecting the sphingolipid biosynthesis pathway. Genetic and pharmacological depletion of serine palmitoltransferase activity, responsible for the first step in sphingolipid biosynthesis, causes a severe polarity defect, thereby suggesting that sphingolipids contribute significantly to hyphal extension [14]. Further evidence that links sphingolipids to sterol-rich domains comes from the functional characterization of the acyl-CoA dependent ceramide synthase BarA in *A. nidulans.* Ceramides are the “simplest” of the sphingolipids, in that they only contain hydrogen as the head group, and they serve as a template for the synthesis of more complex sphingolipids. BarA was originally identified as a gene product that was necessary for sensitivity to the heat-stable antifungal factor (HSAF) from the bacterium *Lysobacter enzymogenes* [15]. HSAF is a mixture of three structurally-related compounds, with dihydromaltophilin as the primary one [16]. The genome of *A. nidulans* encodes at least two ceramide synthases, *barA* and *lagA*. Deletion of BarA function causes a hyphal polarity defect and disrupts the organization of sterol-rich domains at hyphal tips, while deletion of the *lagA* gene did not yield a viable mutant. However, when repressed with an inducible promoter, *lagA*-depleted mutants displayed a severe reduction in growth compared to the *barA* mutant, suggesting that two pools of ceramide are produced in *A. nidulans* and each contributes differentially to fungal growth, with the LagA pool being essential for cell viability [15]. Indeed, two pools of ceramide have been demonstrated in other organisms, and they mainly differ based on the length of their fatty acid chain [17], [18]. Importantly, the pool of ceramide produced by BarA appears to contribute specifically to membrane organization at the hyphal tip and hence to polarized growth [15].

The first objective of this study was to characterize the role of lipid microdomains in the process of host infection by *F*. *graminearum*. Our prior studies suggest that cell surface organization impacts pathogenicity [19]. Here, we exploit our previous work on the *A. nidulans* BarA ceramide synthase to address the role of sphingolipids in this process. In particular, we hypothesized that deletion of the *F*. *graminearum barA* homologue *BAR1* would alter cell surface organization and hence disturb plant infection. The second objective of this study was to determine the nature of the ceramides generated by the BarA/Bar1 ceramide synthase. Given that different classes of ceramides are produced (e.g. different fatty acid chains, different head groups etc.), we surmised that Bar1 contributes to the production of a specific class of sphingolipid.

## Materials and Methods

### Strains and culture conditions

All *F. graminearum* strains used in this study were derived from strain PH-1 (NRRL 31084). The Δ*BAR1* mutants were generated by transforming strain PH-1 as described below. Strain P2 is a derivative of PH-1 that expresses the hygromycin phosphotransferase (hph) gene from plasmid pUCH2-8 [19]. Strains Δ*FMK1* and Δ*MGV1* were the kind gifts of Dr. Jin-Rong Xu, Purdue University. Stocks were maintained by storing mycelia in 30% (v/v) glycerol solution at −80°C. Strains were maintained solid V8 agar medium [19].

To assess macroconidia production, 100 µl of a 1×10^4^ per ml macroconidial suspension was spread inoculated onto YMA [20] or YMA+4% NaCl and incubated at room temperature for seven days. In general, we obtain greater yields of macroconidia when using this approach instead of the standard method of growth in liquid CMC media (also, some of the mutants generated in our laboratory do not sporulate well in CMC). Macroconidia were harvested in 2 ml of sterile distilled water and counted with a hemacytometer. Three readings were recorded per plate and averaged, with the data presented representing averages from five replicate plates, Macroconidia lengths and widths were measured using differential interference contrast microscopy and IPLab Imaging Software (Scanalytics, Inc).

Biomass was assessed by inoculating 50 ml liquid YMA with 5 µl of 1×10^5^ per ml macroconidia suspension, followed by incubation on a rotary shaker set at 28°C and 200 RPM for three days. The resulting mycelium was dried at 60°C for 16 hours and the mass was recorded. Measurements represent the average across three independent experiments.

Wheat head inoculations (variety “Norm”) were performed as previously described [19] . Development of subcuticular and wide, invasive hyphae was observed on detached wheat glumes as previously described [21]. Sexual crosses were performed on carrot agar as previously described [19], [22]. Sensitivity to heat stable antifungal factor [HSAF; 16] was tested by mixing HSAF (suspended in methanol) with molten YMA amended to 0.05% Tergitol. Equal amounts of methanol were added to each treatment group as a control.

### Generation of ΔBAR1 mutant

To identify an *F*. *graminearum barA* homologue, the BarA sequence (ANID_04332; http://www.broadinstitute.org/annotation/genome/aspergillus_group/MultiHome.html) was used in a pBLAST search against the F. graminearum proteome (http://www.broadinstitute.org/annotation/genome/fusarium_group/MultiHome.html). For phylogenetic analysis, the amino acid sequences of other fungal ceramide synthases were retrieved from the NCBI (www.ncbi.nlm.nih.gov). MacVector software (MacVector Inc, Cary, North Carolina) was used to align the sequences and generate a neighbor-joining tree, and bootstrap values were generated after 1000 iterations.

**Figure 1:**
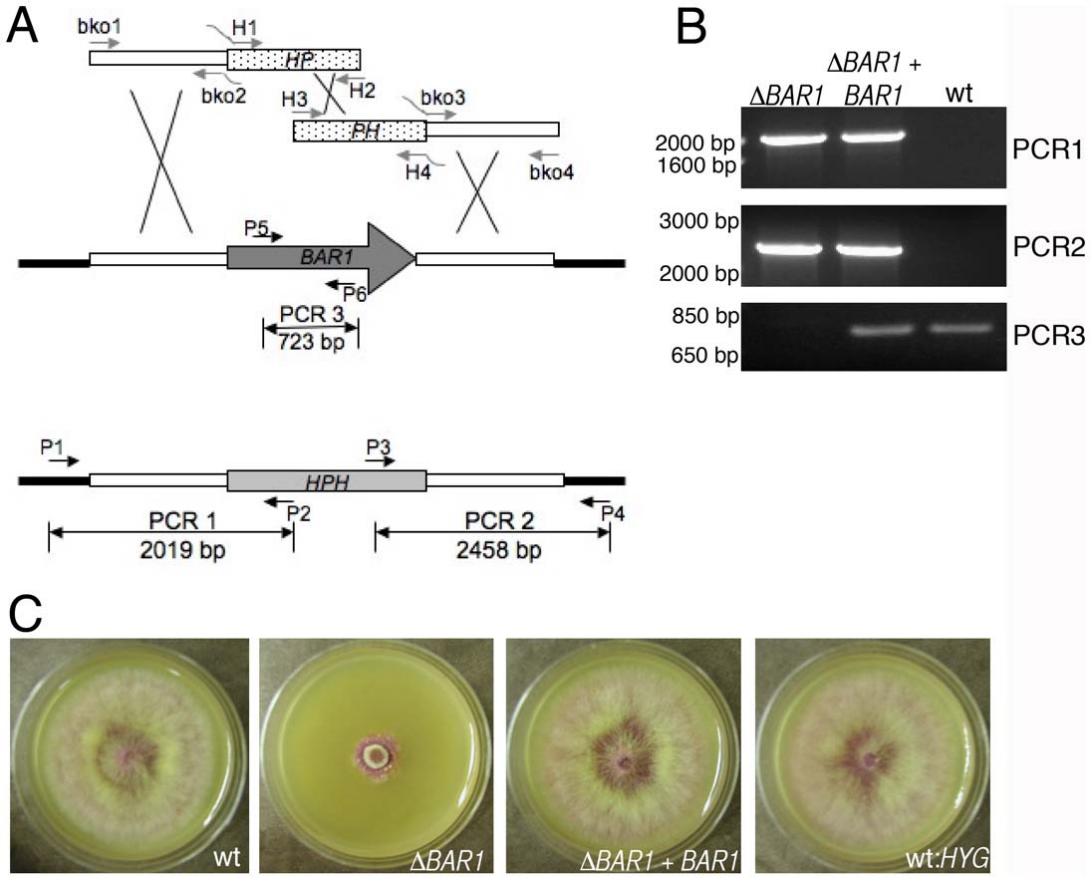
Replacement of the *BAR1* gene with a hygromycin resistant cassette. A. Schematic depiction of the split-marker strategy. White bars represent non-coding genomic DNA on the 5’ and 3’ end of the *BAR1* gene. These regions were amplified and incorporated into the replacement construct to promote homologous recombination. Black bars represent genomic DNA outside of the construct. The designated PCR reactions with primers P1-P6 were used as a diagnostic test for *BAR1* replacement. B. Diagnostic PCR results. Numbers on the left side represent the migration of a standard DNA ladder. C.Δ*BAR1* and control colonies after 4 days growth on V8 medium.

A split-marker approach was used to replace the endogenous *BAR1* (FGSG_09423.3) gene of *F. graminearum* with a hygromycin phosphotransferase (hph) marker from plasmid pUCH2-8 [23]. The constructs necessary to perform this transformation were obtained using the following steps. First, a 1890 bp fragment upstream of *BAR1* start codon was amplified from genomic DNA with primers bko1 and bko2 (Fig. 1A). Similarly, a 1989 bp fragment downstream of *BAR1* was amplified using primers bko3 and bko4. Also, two separate fragments were amplified from the hph cassette of pUCH2-8; the first fragment was amplified with primers H1 and H2 and encompassed the first 1737 bp of the cassette, while the second fragment was amplified with primers H3 and H4 and encompassed the last 1626 bp of the cassette [19]. Importantly, these two fragments from the hph cassette shared 552 bp of homologous sequence [24]. Primers bko2/H1 and primers H4/bko3 had complementary tails to promote fusion PCR (see below). All fragments were then gel purified using the QIAquick Gel Extraction Kit (Qiagen). Then, a fusion PCR approach was used to fuse the upstream genomic fragment to the first hph sequence, and a separate reaction was used to fuse the second hph fragment to the downstream genomic fragment (Fig. 1A). All of the above PCR reactions (including the fusion PCR) were performed using the High Fidelity PCR kit (Roche) according to the manufacturer’s instructions. The template DNA used for the fusion PCR was an equal amount of the two fragments to be fused plus primers bko1/H2 (for the upstream fusion fragment) or primers H3/bko4 (for the downstream fusion fragment).

Replacement of the endogenous *BAR1* gene was accomplished by transforming protoplasts of strain PH-1 with the two fusion fragments (750 ng of each fragment) generated as described above. Transformation of protoplasts was performed as described previously [19]. Hygromycin resistant (hyg^R^) colonies were collected 7–10 days later and cultured on V8 medium with hygromycin B (300 µg/ml). Proper incorporation of the hph cassette at the *BAR1* locus was assessed using primers P1–P6 and Taq polymerase (Invitrogen) according to the manufacturer’s instructions (Fig. 1). The Δ*BAR1*-31 strain was complemented with plasmid pBR30.1, which was generated by ligating a full-length copy of *BAR1* into the XbaI site of plasmid NatXho1-1 containing a nourseothricin resistance marker.

### Characterization of cell surface organization in ΔBAR1 mutants

Nuclei and hyphae were observed in germinated macroconidia by staining with Hoechst 33258 (Molecular probes) and Calcofluor ( = fluorescent brightener 28; Sigma) respectively, as described previously [20]. In order to observe the distribution of sterol-rich lipid microdomains, germinated macroconidia were stained for three minutes in media containing 15 µg/ml Filipin (Sigma) and imaged using an Olympus BX51 fluorescent microscope. To assess cell wall defects, strains were tested on YMA containing Calcofluor white (fluorescent brightener 28; Sigma) and Congo red (Sigma) as described previously [25].

To test for presence of the Spitzenkorper, macroconidia were grown for four hours at 28°C on coverslips covered with liquid YMA. After 4 hours, coverslips were stained with 32 µM FM4-64 (Sigma) and washed 3 times in pre-warmed YMA. The coverslips were then submerged once again into liquid YMA, incubated for 1.5 more hours, then imaged using fluorescence microscopy. All images were recorded within three minutes of mounting. Germinated macroconidia were stained at least two times for each analysis.

### Analysis of sphingoid long chain bases

Long chain bases (LCBs) of sphingolipids were analyzed by hydrolyzing the amide bond between the LCB and the fatty acid using a protocol described previously [26]. The hydrolyzed bases were then conjugated to *o*- phthaldialdehyde and analyzed with high performance liquid chromatography (HPLC) with a C18 column. The production of glucosylceramide was further tested by enriching lipid fractions for glucosylceramides and thin layer chromatography as described previously [26].

### In silico analysis of BAR1 and GCS1 expression

The plant expression database (PLEXdb; http://www.plexdb.org/) was used to obtain expression data of probesets. Probeset annotations (fgd384–480 = BAR1; fgd166–410 = FGSG_03851, fgd237–640 = GCS1) were obtained from MIPS Fusarium graminearum Genome Database (http://mips.helmholtz-muenchen.de/genre/proj/FGDB/). Expression was assessed during barley invasion, conidial germination, and sexual development.

Generation of the Yarrowia lipolytica ΔGCS1 mutant.

The *URA3* gene (*YALI0E26741g*) of *Y. lipolytica* was used for targeted integrative disruption of the *GCS1* (*YALI0B09669g*) gene. A ∼1.0 kb DNA fragment upstream of the ATG start codon of the *GCS1* open reading frame was amplified by the PCR using the primers *GCSHind* III-A and *GCSBamH*I-B. The resulting fragment was digested with *Hind* III and *BamH*I and inserted into the corresponding sites of the vector pUC19 to generate pUC19UP. Then a ∼1.0 kb DNA fragment downstream of the stop codon of the open reading frame was amplified using the primers *GCSBamH*I-C and *GCSEcoR*I-D. This fragment was digested with *BamH*I and *EcoR*I and inserted into the vector pUC19UP to produce the vector pUC19UPDS. Then a ∼4.8 Kb DNA fragment containing the *Yarrowia lipolytica URA3* gene was amplified with the primers *URA*5’ and *URA*3’. This fragment was digested by *BamH*I and ligated into the *BamH*I site of the vector pUC19UPDS. Then a ∼6.8 Kb DNA fragment containing the *URA3* gene flanked by the upstream and downstream of the *GCS* sequence was amplified by primes *GCSHind* III-A and *GCSEcoR*I-D. The PCR product was gel purified and used to transform *Y. lipolytica* strain ATCC90811 (*leu2-35 lys5-12 ura3-18*) using the frozen-EZ yeast transformation II kit ( Zymo Research, Orange, CA). Transformants were selected on URA dropout media [0.17% (w/v) yeast nitrogen base, 2% (w/v) glucose, 0.5% (w/v) ammonium sulfate, and 0.08% (w/v) CSM-URA (MP Biomedicals, Solon, OH)]. Genomic DNA obtained from transformants was analyzed by PCR to confirm deletion. For this analysis, the primers *GCS*up (located ∼1.1 Kb upstream of the GCS ATG start codon) and *GCS*down (located internal of the DNA fragment containing the *URA3* genomic gene) were used. Potential deletion mutants were further confirmed by thin layer chromatographic analysis of lipid extracts as previously described [26].

## Results

### Identification and deletion of BAR1 in Fusarium graminearum

We have previously described the existence of two distinct clades of acyl-CoA-dependent ceramide synthases in filamentous fungi [15]. This analysis revealed the presence of ceramide synthases related to both LagA (FGSG_05525) and BarA (FGSG_09423 and FGSG_03851) in *F*. *graminearum*. Here, we present the characterization of FGSG_09423, now designated Bar1, which possesses 41% identity and 58% similarity (E-value = 1e^−92^) with BarA. We note that FGSC_03851, which has slightly weaker homology to BarA (39% identity, 58% similarity, E-value = 2e^−76^), will be the subject of future studies.

The expression of the *BAR1* and FGSG_03851 genes remains relative unchanged during *F*. *graminearum* infection of barley as well as during nutrient starvation. However, whereas the *BAR1* transcript increases approximately 10-fold during the first 2 hours after macroconidia germinate, the FGSG_03851 transcript decreases approximately 4 orders of magnitude during this same time period (Fig. S1). On the other hand, *BAR1* and FGSG_03581 are expressed at comparable levels during latter stages of *in vitro* growth (i.e., 72–96 hrs.) as well as during in vivo sexual development (dikaryotic hyphae to mature perithecia).

In order to characterize the function of Bar1 in *F. graminearum*, a split-marker approach was used to replace the *BAR1* gene in wild-type strain PH-1 with a hygromycin phosphotransferase marker from plasmid pUCH2-8. Various primers were designed to confirm the complete replacement of the endogenous *BAR1* gene with the hph marker (Fig. 1). Two independent transformants were collected and displayed similar phenotypes (strains Δ*BAR1*-21 and Δ*BAR1*-31). Δ*BAR1* phenotypes (see below) were complemented by transforming the Δ*BAR1*-31 strain with plasmid pBR30.1, which contains an endogenous *BAR1* coding sequence plus a nourseothricin-resistance maker (Fig. 1C).

### Growth and sporulation in ΔBAR1 mutant

**Figure 2:**
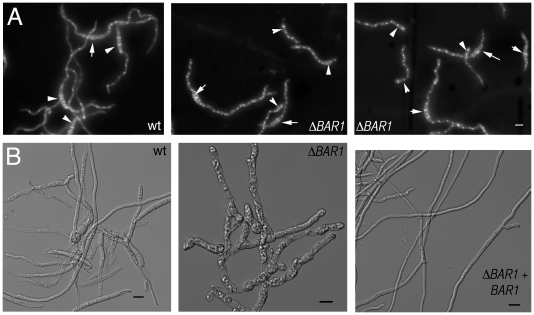
Spore germination and deterioration in Δ*BAR1* mutants. A. Germinating macroconidia (white arrows) 8 hours post inoculation. Stained with Calcofluor white (cell wall) and Hoechst (nuclei). Germination and cell cycle appears normal in Δ*BAR1* mutant, as they are capable of releasing a germ tube and duplicating nuclei. All micrographs at the same scale for panel A. Scale bar = 10 µm. B. Germinated spores after 4 days of incubation in liquid CMC medium. Note the abnormal morphology displayed by the Δ*BAR1* mutant. Scale bar = 10 µm.

Δ*BAR1* mutants displayed a severe colonial phenotype (Table 1; Fig. 1C). In addition, the biomass of strain Δ*BAR1*-31 was lower than that of the wild-type and ectopic strain after three days of growth in liquid YMA (Table 1). Nevertheless, macroconidia of Δ*BAR1* germinated at similar time points compared to control strains (Fig. 2A). These data suggest that deletion of *BAR1* triggers growth defects at later stages of colony formation. Accordingly, whereas germlings of Δ*BAR1* mutants looked ‘healthy’ immediately after germ tube release, they were heavily vacuolated and abnormal in appearance upon extended incubation in liquid media (Fig. 2B). One potential explanation for this observation is that accumulation of a metabolite was inducing cell death (i.e. growth rate was hindered only after a period of time that allowed accumulation of a deleterious metabolite). In *A. nidulans*, the sphingoid ceramide precursors dihydrosphingosine and phytoshphingosine induce programmed cell death [PCD]; [27]. In order to determine if PCD was occurring in the Δ*BAR1*-31 strain, germinated macroconidia were stained with an *in situ* cell death detection kit (i.e. TUNEL assay; Roche). While some nuclei of the Δ*BAR1* mutants stained positive, the absence of extensive staining (e.g., as we previously observed in wild-type *F*. *graminearum* hyphae exposed to farnesol; [28]) implies that PCD is not the cause of cell deterioration (data not shown).

**Table 1:** Phenotypic data for the Δ*BAR1* mutant.

Strain	Colony Diameter^1^, cm (SD)	Dry Biomass^2^, mg (SD)	Macroconidia Length^3^, µm (SD)	% Subcuticular hyphae^4^ 24/48 hr	% Bulbous infection hyphae^5^ 24/48 hr
*ΔBAR1*	1.7 (0.10)	158.3 (25.3)	14.7 (2.9)	100/100	97/100
Δ*BAR1*+*BAR1*	4.9 (0.06)	264.7 (4.9)	33.5 (5.1)	ND	ND
wt	4.8 (0.06)	ND	32.4 (6.0)	100/100	100/100
wt:hyg	4.8 (0.06)	275.7 (9.3)	35.0 (6.0)	100/100	100/100

1 -Average diameter of three colonies after 4 days growth on V8 medium, room temperature.

2 -Dry biomass of mycelia after 3 days growth in liquid YMA.

3 -mean length of at least 50 macroconida per strain.

4 -% of glumes, out of 30 inoculated glumes, that had subcuticular hyphae after either 24 or 48 hours.

5 -% of glumes, out of 30 inoculated glumes, that had bulbous infection hyphae in glume epidermal cells after either 24 or 48 hours.

**Figure 3:**
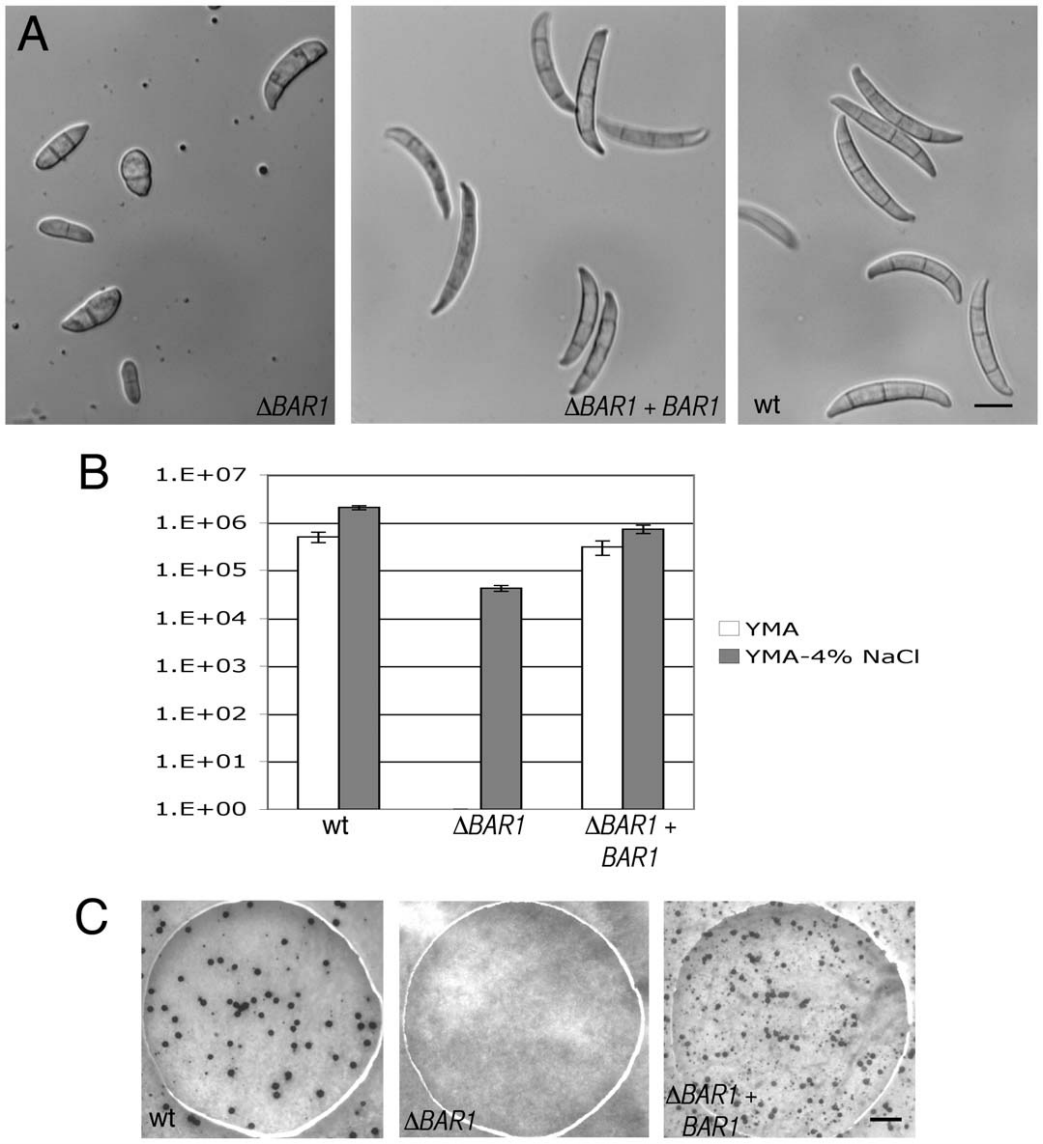
Asexual and sexual reproduction in the Δ*BAR1* mutant. A. Representative spore morphology of Δ*BAR1* and control strains. Scale bar = 10 µm for all micrographs in panel A. B. Macroconidia formation is drastically reduced in Δ*BAR1* strain, a defect which can be partially relieved with addition of 4% NaCl. Note the logarithmic scale. C. Deletion of *BAR1* completely abolishes production of perithecia (black structures). Scale bar = 1 mm for all micrographs in panel C.

While attempting to harvest macroconidia for microscopic observation, it was noticed that Δ*BAR1* mutants sporulate very poorly. No macroconidia were observed when strains were grown on solid YMA or liquid CMC (Fig. 3B; data not shown). This sporulation defect was partially alleviated by amending YMA with 4% NaCl (Fig. 3A,B). Even in this case, the macroconidia of Δ*BAR1* strains displayed morphological abnormalities in that they were significantly shorter and did not display the slender, canoe-shape morphology typical of *F. graminearum* macroconidia (Fig. 3A; Table 1). In addition to the defect in asexual reproduction, the Δ*BAR1* mutant was not capable of forming perithecia, the sexual fruiting structures of *F. graminearum* (Fig. 3C). These data suggest that Bar1 contributes significantly to sexual and asexual sporulation in *F. graminearum*.

**Figure 4:**
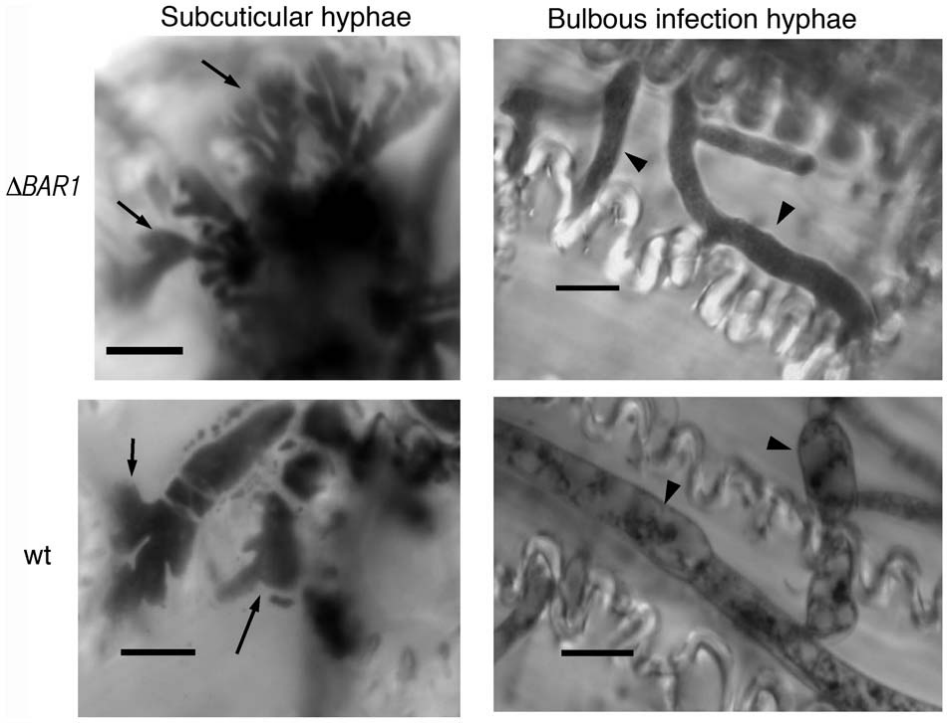
Development of infection-related structures in the ΔBAR1 mutant. Deletion of *BAR1* does not affect the ability to differentiate subcuticular (arrows) or bulbous infection hyphae (arrowheads). Scale bar = 10 µm.

TheΔ*BAR1*-31 strain showed no symptoms when inoculated onto wheat heads (unpublished data). However, the severe growth phenotype exhibited by Δ*BAR1* strains made the interpretation of this result difficult, as any defect in host tissue colonization could not be separated from its severe *in vitro* phenotype. However, the morphological defects displayed in sexual and asexual reproduction made us question whether or not the Δ*BAR1* mutants would be defective in differentiating infection-related hyphae [21]. Despite their inability to efficiently differentiate sexual and asexual structures, Δ*BAR1* mutants were able to differentiate both subcuticular hyphae and bulbous infection hyphae when inoculated onto detached wheat glumes (Fig. 4; Table 1). These data suggest that Bar1 is not absolutely required for infection-related hyphal development in *F. graminearum*.

### Sphingolipid production

**Figure 5:**
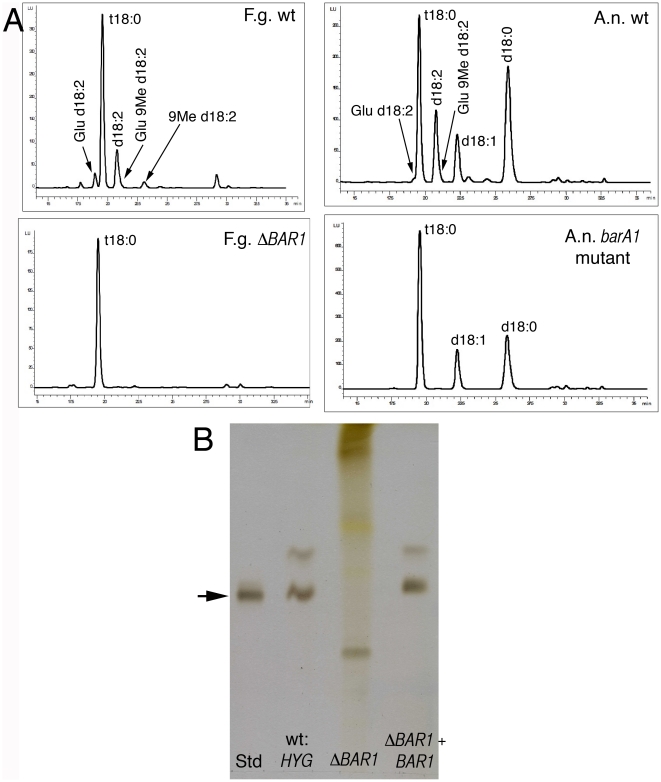
Deletion of *BAR1* causes loss of glucosylceramide production. A. HPLC analysis of o-phthaldialdehyde/long chain base derivatives from wild-type and *BAR1* mutants in both *F. graminearum* (F.g.) and *A. nidulans* (A.n.). ‘X:#’ refers to the hydroxylation status (X) and carbon number (#) of the long chain base. For X, ‘t’ = trihydroxy and ‘d’ = dihydroxy. Glu = glucose. 9Me = methyl group attached to C9 of the long chain base. B. TLC plate of lipid fractions enriched for glucosylceramides. Std = glucosylceramide standard from soybean. The arrow depicts the migration of glucosylceramide (GlcCer).

Ceramides are required for a number of cellular processes, including signaling and membrane integrity [10], [29]. Our previous analyses suggested that polarized hyphal extension may depend upon two distinct pools of ceramide; one generated by BarA, and the other by LagA. Notably, many fungi are capable of producing both inositolphosphoceramides and glucosylceramides [17], leading to the idea that each type is specifically synthesized by one of the ceramide synthases. To better understand the nature of the ceramide pools generated by BarA homologues, *o*-phthaldialdehyde derivatives of the long chain sphingoid bases from the Δ*BAR1* mutant were analyzed by high performance liquid chromatography (HPLC) [30]. Preliminary data suggested that the Δ*BAR1* mutant of *F. graminearum* and the *barA1* mutant of *A. nidulans* were specifically defective in glucosylceramide (GlcCer) production (Fig. 5A). In order to further test this hypothesis, lipid fractions were isolated from *F. graminearum* strains, enriched for GlcCer, and analyzed by TLC. These analyses confirmed that the Δ*BAR1* mutant fails to generate GlcCer (Fig. 5B).

### HSAF sensitivity

**Figure 6:**
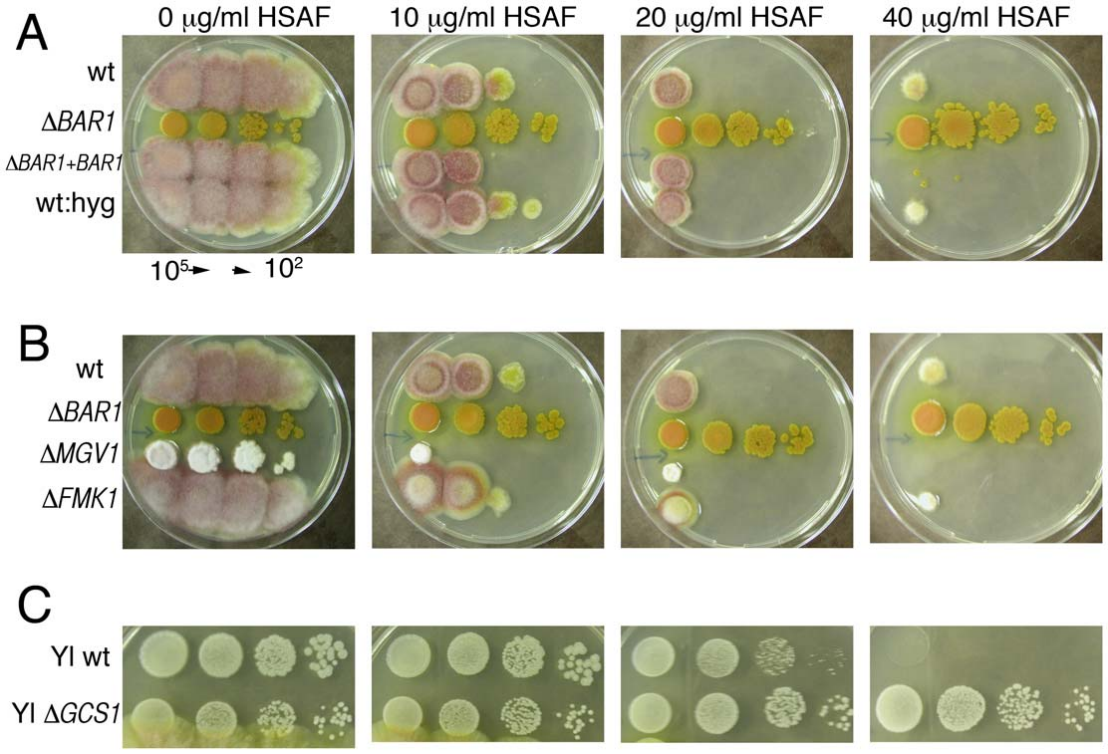
HSAF sensitivity of Δ*BAR1*, Δ*GCS1*, and ΔMAPK mutants. A. Δ*BAR1* mutants are resistant to HSAF. 7 µl of macroconidial suspensions (serially diluted from 10^5^ to 10^2^ per ml) were spotted onto plates containing different concentrations of HSAF. B. MAPK mutant Δ*MGV1* is hypersensitive to HSAF, but Δ*GPMK1* is unchanged in sensitivity. C. Glucosylceramides contribute to HSAF sensitivity, as a *Yarrowia lipolytica* Δ*GCS1* mutant is resistant to HSAF. Plates in panels B and C were inoculated as described in A.

The *barA1* mutation in *A. nidulans* was originally identified as a mutation that conferred resistance to HSAF [15]. Similarly, Δ*BAR1* mutants of *F. graminearum* are resistant to HSAF (Fig. 6A). An *F*. *graminearum* mutant defective in GlcCer production conferred resistance to the plant defensins MsDef1 and RpAFP2 [26]. Since Bar1 is necessary for the production of glucosylceramides, we hypothesized that HSAF may share the same or similar targets as these plant defensins. Accordingly, a readily available Δ*GCS* (glucosylceramide synthase) mutant of the dimorphic fungus *Yarrowia lipolytica* (M. Chen and E. Cahoon, unpublished results) was tested for sensitivity to HSAF. Plating assays indicated that GlcCer is required for HSAF sensitivity, as the Δ*GCS1* mutant is resistant to HSAF (Fig. 6C). The ability of *F. graminearum* to cope with MsDef1 is mediated via the Gpmk1 and Mgv1 MAPKs, as mutants in these two genes are more sensitive to this defensin [31]. To test the possibility that these two MAPKs are also involved in the response to HSAF, the Δ*GPMK1* and Δ*MGV1* mutants were tested for their sensitivity. The Δ*MGV1* mutant exhibited an increased susceptibility to HSAF, whereas the sensitivity of the Δ*GPMK1* mutant was similar to that of wild-type strain PH-1 (Fig. 6B). These data suggest that HSAF and the plant defensins MsDef1/RpAFP2 potentially target fungi via related mechanisms that involve glucosylceramide.

### Cell surface organization in the ΔBAR1 mutant

**Figure 7:**
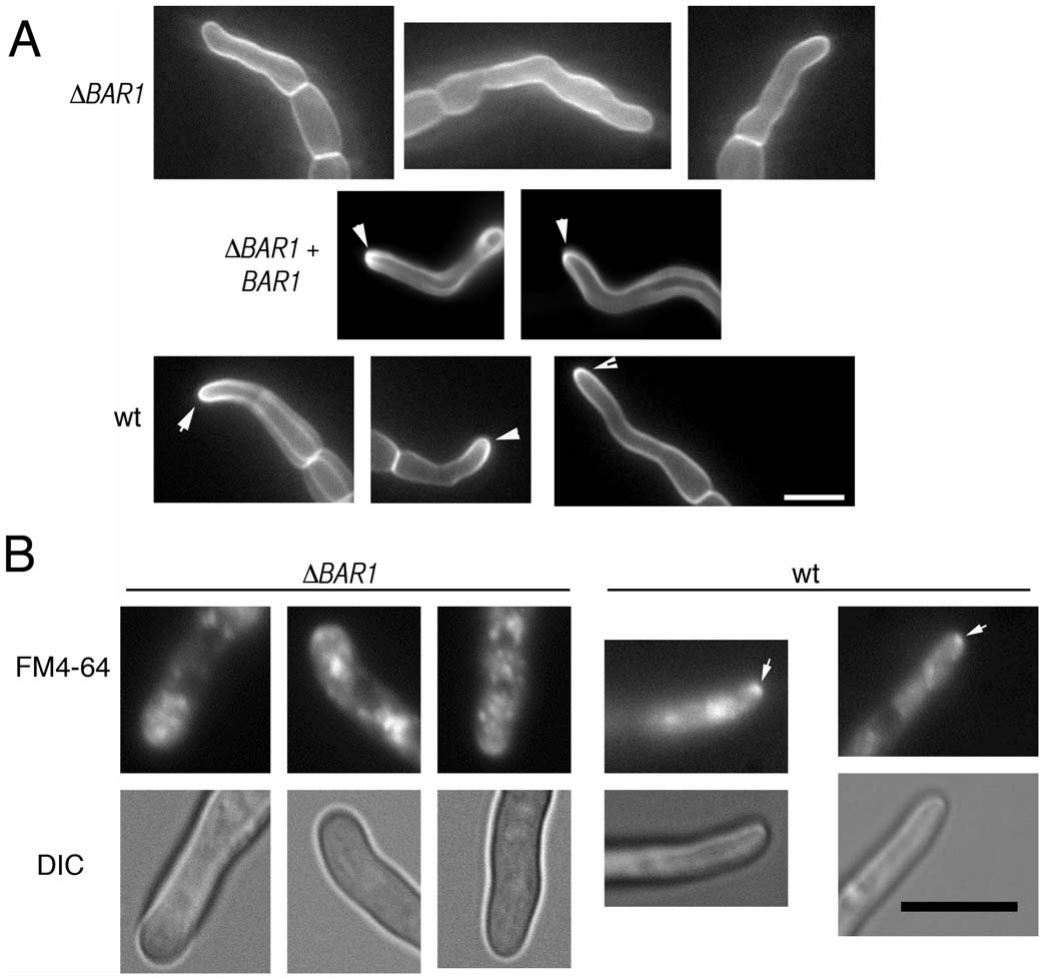
Deletion of *BAR1* causes disruption in hyphal tip organization. A. Representative images of sterol-rich domains stained with filipin at the extreme apex of hyphae. Note the bright regions stained at the tips of wt and complemented strains (arrows) but missing from Δ*BAR1* hyphae. All micrographs in panel A at the same scale. B. Endomembrane systems stained with FM4-64. Wild-type germlings exhibited bright spots at the extreme hyphal apex coinciding with the vesicle-rich Spitzenkörper (arrows). Staining at the hyphal tip of Δ*BAR1* germlings failed to display a discrete Spitzenkörper. All micrographs in panel B at the same scale. Scale bars = 10 µm.

In *A. nidulans*, *barA1* mutants display altered pattern of filipin staining, suggesting a defect in organizing sterol-rich domains at the hyphal tip [15]. Accordingly, *F. graminearum* Δ*BAR1* mutants were examined and found to display a similar pattern of filipin localization, in which hyphal tips did not stain brighter compared to distal sections of germ tubes (Fig. 7A). Also, the germ tubes of Δ*BAR1* mutants were wider and failed to consistently display a Spitzenkorper when stained with FM4-64 (Fig. 7B). These data suggest that Bar1 is essential for maintaining a defined polarity axis during hyphal growth.

In addition to organization at the membrane surface, we also hypothesized that Δ*BAR1* mutants would exhibit cell wall defects. Sterol-rich domains may represent membranous areas that are rich in lipid microdomains, which have been shown to aggregate membrane proteins such as glycosylphosphatidylinositol (GPI)-anchored proteins [8], [10]. Whereas GPI-anchored proteins are exclusively membrane proteins in other kingdoms, fungi have the ability to cleave them from the membrane and covalently attach them to the carbohydrate backbone of cell walls [32]. Therefore, a defect in membrane organization may also result in a defect in cell wall organization. To test for cell wall defects, Δ*BAR1* mutants were grown on media containing calcofluor white and Congo red. Calcofluor white and Congo red are two compounds that bind to chitin in fungal cell walls and are useful indicators of cell wall defects [25]. The Δ*BAR1* mutant was more sensitive to both compounds, whereas PH-1 and the complemented mutants were resistant, even up to 10 mg/ml for Congo red (Fig. S2; data not shown). These data suggest that Bar1 contributes to the cell wall structure of *F. graminearum*, Nevertheless, TEM analysis failed to identify any gross structural changes in the cell wall of Δ*BAR1* mutants (Fig. S2).

## Discussion

Previous work has implicated fungal acyl coA-dependent ceramide synthases of the BarA class in the regulation of polarized hyphal growth. Here, we present the first characterization of BarA in a plant pathogenic fungus, with a view toward determining its importance for virulence. We also describe biochemical experiments designed to reveal the nature of the ceramides generated by BarA. Our results demonstrate that the *F*. *graminearum* BarA homologue, Bar1, plays an unexpectedly significant role in growth and development. In addition, we find that BarA homologues from *F*. *graminearum* and *Aspergillus* specifically generate glucosylceramides. Collectively, our results emphasize the importance of glucosylceramides as key regulators of growth and development in filamentous fungi.

### Roles of Bar1 in growth, development, and pathogenicity

Unlike *A. nidulans*, *F*. *graminearum* possesses two BarA homologues; FGSG_09423 (Bar1) and FGSG_03851 [15]. Because Bar1 possesses slightly greater homology to *A. nidulans* BarA, and is expressed at much higher levels during the period following spore germination, our initial efforts focused on its characterization. Surprisingly, deletion of *BAR1* caused significant defects in growth, formation of macroconidia, and sexual development. By contrast, in *A*. *nidulans*, null *barA* mutants are capable of forming relatively normal sporulating colonies despite defects in maintaining axes of hyphal polarity [15]. Furthermore, defects caused by the absence of Bar1 in *F. graminearum* only became apparent several hours after spore germination. The abnormal appearance of these “older” hyphae raised the possibility that they had undergone PCD, but no evidence for apoptosis could be detected using TUNEL assays. Although poor growth of the Δ*BAR1* mutant could reflect the accumulation of a toxic metabolite that triggers a necrotic response, an alternative explanation is that Bar1 is required for hyphal maturation. In other filamentous fungi (i.e., *Ashbya gossypii* and *A. nidulans*), growing germlings mature into hyphae that extend at a much faster rate via a mechanism that requires microtubules and PAK kinases [33], [34]. In Δ*BAR1* mutants, failure of hyphae to mature may lead to slow growth and accumulation of vacuoles.

When tested using a detached wheat glume assay, Δ*BAR1* mutants were able to differentiate both subcuticular hyphae and bulbous infection hyphae. Accordingly, the early stages of infection-related morphogenesis do not appear to require functional BarA. Nevertheless, Δ*BAR1* mutants did not trigger any symptoms when inoculated onto wheat heads. This observation is presumably a reflection of the overall growth defect caused by absence of BarA, as opposed to a specific role in pathogenesis. Results from other studies, however, do implicate glucosylceramides as important factors in the virulence of both plant and animal pathogens [26], [35]. Notably, glucoylceramides were recently identified as important virulence factors in the human pathogen *Candida albicans* despite the absence of any overt role in hyphal morphogenesis [36].

The severity of the phenotypes caused by deletion of *BAR1* was somewhat surprising given the presence of a second homologue, FGSG_03851. The relatively poor expression of this homologue during hyphal growth implies that Bar1 is the dominant ceramide synthase of this class during this phase of the life cycle. On the other hand, FGSG_03851 is expressed at the same levels as Bar1 during sexual development, yet the *BAR1* deletion still confers a significant developmental defect. This observation suggests that these two BarA homologues do not simply function redundantly. One interesting possibility is that FGSG_03851 might be associated with a toxin biosynthetic cluster. Indeed, the predicted functions of nearby genes coincide with the functions of many mycotoxin biosynthesis genes (Tri8 orthologue, p450 oxidases, transcription factor etc; Table S1). Also, in other *Fusarium* species (i.e., *F. verticillioides*), BarA homologues (i.e., Fum18) flank the fumonisin biosynthetic gene cluster [37], where they may play a role in altering plant or fungal ceramide pools in the presence of fumonisins.

### BarA homologues generate glucosylceramides

Our prior studies in *A. nidulans* strongly suggested the presence of two distinct ceramide pools that regulate growth and morphogenesis in filamentous fungi [15], [38]. In particular, BarA was proposed to generate a specialized ceramide pool that ensures the maintenance of hyphal polarity, whereas LagA is responsible for bulk ceramides that contribute to growth. Here, our analysis of sphingolipids in both *A. nidulans* and *F. graminearum* establish that BarA homologues generate C18 glucosylceramides. Furthermore, the phenotypes of *barA* and Δ*BAR1* mutants implicate glucosylceramides as important regulators of hyphal morphogenesis and development. For example, they may help to promote the formation of membrane domains at hyphal tips that recruit the Spitzenkorper and other components needed for rapid hyphal extension.

**Figure 8:**
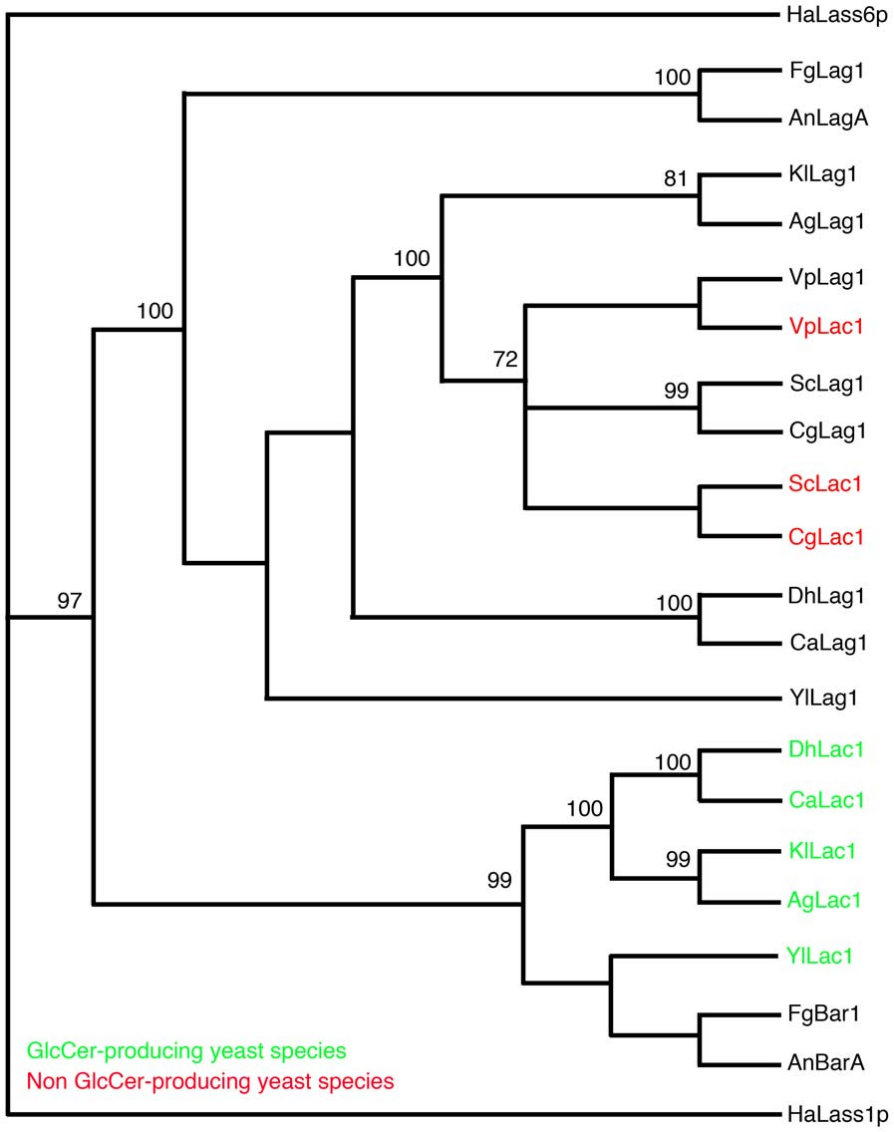
Neighbor-joining phylogenetic analysis of Lag1, Lac1, and Bar1 protein sequences in *Ashbya gossipii* (Ag), *A. nidulans* (An), *Candida albicans* (Ca), *Candida glabrata* (Cg), *Debaryomyces hansenii* (Dh), *F. graminearum* (Fg), *Kluyveromyces lactis* (Kl), *Vanderwaltozyma polyspora* (Vp), and *Yarrowia lipolytica* (Yl). HsLass1p/6p are ceramide synthase sequences from *Homo sapiens* used as out-groups. This analysis suggests that ScLac1 (and Lac1 genes from other non GlcCer-producing yeast strains) likely represents a Lag1 *para*log, whereas Lac1 from GlcCer-producing yeast strains is an ancestral Bar1 *ortho*logue that was lost in non GlcCer-producing strains. Bootstrap values calculated from 1000 iterations.

The non-glucosylceramide-producing yeast *S. cerevisiae* encodes two ceramide synthases, *lac1* and *lag1*, which are functionally redundant and prefer C:26 fatty acids as a substrate for ceramide production [39], [40]. Ceramides with C:26 fatty acids are ultimately converted to inositol phosphorylceramides that serve several essential functions in cells. However, in the glucosylceramide-producing yeast species *Kluyveromyces lactis*, Lac1 is specifically necessary for the production C:18 sphingolipids, including glucosylceramide [17]. Phylogenetic analysis of Lag1 and Lac1 amino acid sequences among several yeast species revealed the Lag1 and Lac1 from non-producing strains cluster together into a single clade, whereas Lag1 and Lac1 sequences from glucosylceramide-producing strains separate into two distinct clades (one ‘Lag’ clade and one ‘Lac’ clade) [17]. These data suggest the Lag1 and Lac1 in non-producing strains are actually paralogous, that is, they represent duplicates of an ancestral gene ( = *lag1*). In contrast, Lac1 proteins in glucosylceramide-producing strains likely represent orthologs of an ancestral ceramide synthase distinct from Lag1 (i.e. presumably BarA). Accordingly, we speculate that ‘Lac1’ proteins in glucosylceramide-producing yeast strains are actually Bar1 orthologues (which have been lost in non GlcCer-producing strains) required for production of C:18 ceramides and ultimately, as demonstrated in this study, glucosylceramides. Phylogenetic analyses support this hypothesis, as *F. graminearum* Bar1 and *A. nidulans* BarA form a distinct clade with ‘Lac1’ proteins from glucosylceramide-producing yeasts, while Lac1 proteins from non-producing strains cluster within the Lag1 clade (Fig. 8).

### Glucosylceramides as targets for anti-fungal compounds

Like its *A. nidulans* orthologue BarA, Bar1 is required for sensitivity to the antifungal HSAF. The observations that Δ*BAR1* mutants are resistant to HSAF and do not produce glucosylceramides raises the possibility that HSAF targets these sphingolipds. Consistent with this notion, HSAF is ineffective against *S. cerevisiae*, which does not typically generate glucosylceramide [18], [41]. Further support for this notion was demonstrated in this study, as a glucosylceramide synthase (*GCS*) deletion mutant in the dimorphic yeast *Yarrowia lipolytica* is resistant to HSAF. Interestingly, glucosylceramides have been implicated in the sensitivity to other antifungal compounds. For example, Δ*GCS1* mutants of *F. graminearum* are resistant to the plant defensins MsDef1 and RpAFP2 [26]. The interactions of these defensins with glucosylceramide may be direct, as the defensin RpAFP2 was shown to directly bind to glucosylceramides of fungal origin [42]. *F. graminearum* MAPK mutants Δ*MGV1* and Δ*GPMK1* are hypersensitive to MsDef1, suggesting that their signaling cascades mediate basal resistance to this plant defensin [31]. Similarly, Δ*MGV1* mutants are more sensitive to HSAF, whereas Δ*GPMK1* mutants do not differ from wild type in their sensitivity. Our data illustrate similarities, but also differences, between how HSAF and MsDef1 target fungal cells, though both clearly involve glucosylceramide.

### Conclusions

We have demonstrated that the *BAR1/barA* ceramide synthase genes *in F. graminearum* and *A. nidulans* are specifically required for the production of glucosylceramides. Their failure to generate this class of ceramides is likely responsible for their resistance to HSAF, as a *Y. lipolytica* Δ*GCS1* mutant is also resistant to HSAF. The failure to produce glucosylceramides, coupled with the fact that C:18-ceramide is the typical substrate from which glucosylceramides are synthesized, suggest that Bar1 and its orthologs specifically catalyze the condensation of C:18 fatty acids with sphingoid bases. Interestingly, the Bar1 enzyme appears to have been lost in some yeast species and replaced with a duplicate copy (i.e. paralog) of the C:26-ceramide producing Lag1. Presently, the ‘non-Lag’ ceramide synthases of several yeast species are designated ‘Lac1’, though they more closely resemble Bar1 orthologs (Fig. 8). Our data also provide some preliminary evidence as to the role that glucosylceramides (or other C:18-ceramides) may play in filamentous fungi, such as their importance in sexual reproduction and cell wall organization. Future work should focus on the specific function of C:18-ceramides at cell surface organization and vesicle transport at the hyphal tip.

## Supporting Information

Figure S1 **Expression of ceramide synthases in** ***F. graminearum*** **.** Transcript levels of *BAR1* and FGSG_03851 during conidia germination (A), *in vitro* development (B), and *in planta* development (C). In panel A, x-axis = hours of germination. In panel B, x-axis = hours after perithecia induction. In panel C, x-axis = developmental stage: IF = narrow hyphae; RW = wide, dikaryotic hyphae; SW = perithecia initials; YP = young perithecia. All expression data obtained from the Barleybase website (http://www.plexdb.org/modules/PD_browse/experiment_browser.php). The values are shown from each of three replicates.
(TIF)

Figure S2 **Deletion of** ***BAR1*** **results in cell wall defects.** A. Sensitivity of Δ*BAR1* mutant to the cell wall-perturbing agent Calcofluor white (CFW). B. Transmission electron micrographs of hyphae. The Δ*BAR1* mutant appears to have a seemingly intact outer protein layer (black arrows). However, white arrows indicate electron-dense aggregates that accumulated on the intracellular face of the cell wall of Δ*BAR1* mutants. Scale bar = 500 nm.
(TIF)

Table S1 **Genes flanking FGSG_03851 and predicted function of corresponding proteins.**
(DOC)
