# Supplementary figures and images for: Control of Glucosylceramide Production and Morphogenesis by the Bar1 Ceramide Synthase in Fusarium graminearum
Source: PLoS One. 2011 Apr 29;6(4):e19385. doi: 10.1371/journal.pone.0019385 (PMC3084840; doi:10.1371/journal.pone.0019385)

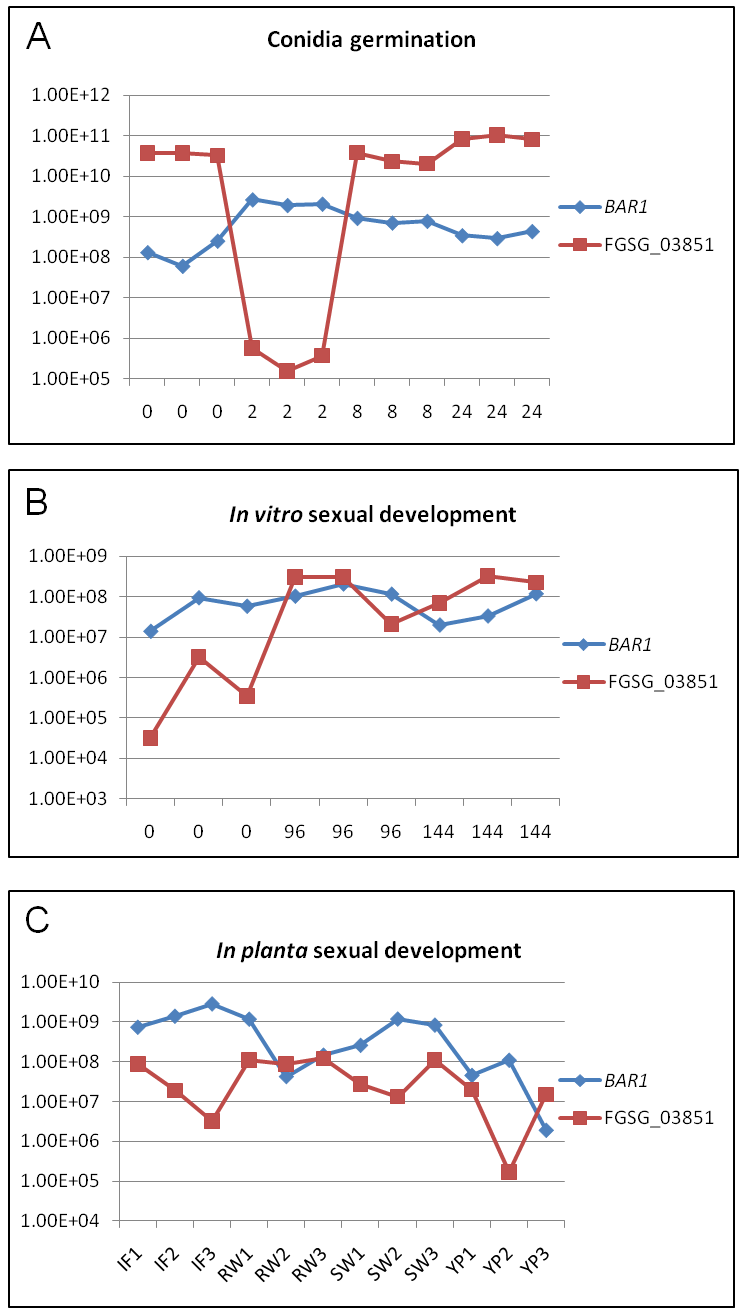

Supplement: Figure S1 — Expression of ceramide synthases in F. graminearum . Transcript levels of BAR1 and FGSG_03851 during conidia germination (A), in vitro development (B), and in planta development (C). In panel A, x-axis = hours of germination. In panel B, x-axis = hours after perithecia induction. In panel C, x-axis = developmental stage: IF = narrow hyphae; RW = wide, dikaryotic hyphae; SW = perithecia initials; YP = young perithecia. All expression data obtained from the Barleybase website (http://www.plexdb.org/modules/PD_browse/experiment_browser.php). The values are shown from each of three replicates. (TIF) [file pone.0019385.s001.tif]

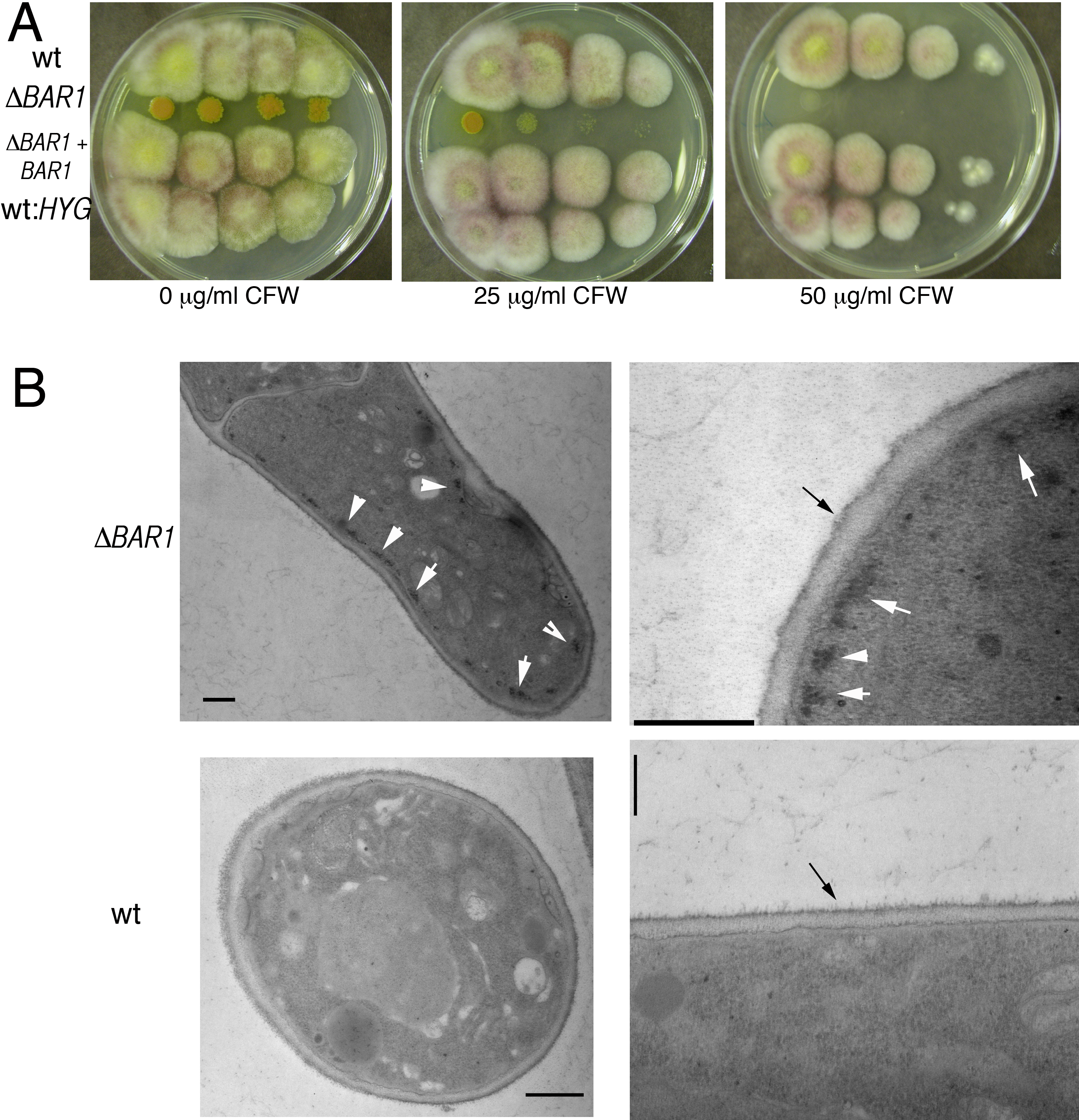

Supplement: Figure S2 — Deletion of BAR1 results in cell wall defects. A. Sensitivity of ΔBAR1 mutant to the cell wall-perturbing agent Calcofluor white (CFW). B. Transmission electron micrographs of hyphae. The ΔBAR1 mutant appears to have a seemingly intact outer protein layer (black arrows). However, white arrows indicate electron-dense aggregates that accumulated on the intracellular face of the cell wall of ΔBAR1 mutants. Scale bar = 500 nm. (TIF) [file pone.0019385.s002.tif]
