# Supplementary material for: Control of Glucosylceramide Production and Morphogenesis by the Bar1 Ceramide Synthase in Fusarium graminearum
Source: PLoS One. 2011 Apr 29;6(4):e19385. doi: 10.1371/journal.pone.0019385 (PMC3084840; doi:10.1371/journal.pone.0019385)
Supplement: Table S1 — Genes flanking FGSG_03851 and predicted function of corresponding proteins. (DOC) [file pone.0019385.s003.doc]

**Table S1.** Genes flanking FGSG_03851 and predicted function of corresponding proteins.

|  |  |  |
| --- | --- | --- |
| **Accession #** | **Strand** | **Predicted Function^1^** |
| FGSG_12374 | + | chromate transporter |
| FGSG_12373 | + | FAD dependent oxidoreductase |
| FGSG_03844 | + | MFS multidrug transporter |
| FGSG_03845 | + | NAD dependent epimerase/dehydratase |
| FGSG_03846 | - | Tri8 Orthologue (FGSG_03532) |
| FGSG_03847 | + | Transmembrane protein 14 |
| FGSG_03848 | - | Ras-like GTPase superfamily |
| FGSG_03849 | + | no predicted function |
| FGSG_12372 | - | p450 Superfamily |
| FGSG_12371 | - | p450 Superfamily |
| FGSG_12370 | - | F-box domain containing protein |
| FGSG_12369 | - | peroxidase/catalase |
| FGSG_03851 | - | BarA orthologue |
| FGSG_12368 | - | zinc binding dehydrogenase |
| FGSG_12367 | - | no predicted function |
| FGSG_03853 | + | short chain dehydrogenase |
| FGSG_12366 | + | glutamate synthase |
| FGSG_03854 | - | condensation superfamily; alcohol acetyltransferases |
| FGSG_03855 | - | MFS; panthothenate transporter |
| FGSG_03856 | - | peptidase; amidohydrolase |
| FGSG_03857 | + | Zn(II)2Cys6 transcription factor |
| FGSG_03858 | - | Cel3e putative secreted beta-glucosidase |
| FGSG_03859 | + | no predicted function |
| FGSG_03860 | + | p450 Superfamily |

**^1^** – protein sequences were downloaded from [www.broad.mit.edu](http://www.broad.mit.edu/) and used in a blastp search against the NCBI non-redundant protein database (www.ncbi.nlm.nih.gov)
